# Taxonomic identification accuracy from BOLD and GenBank databases using over a thousand insect DNA barcodes from Colombia

**DOI:** 10.1371/journal.pone.0277379

**Published:** 2023-04-24

**Authors:** Nathalie Baena-Bejarano, Catalina Reina, Diego Esteban Martínez-Revelo, Claudia A. Medina, Eduardo Tovar, Sandra Uribe-Soto, Jhon Cesar Neita-Moreno, Mailyn A. Gonzalez

**Affiliations:** 1 Instituto de Investigación de Recursos Biológicos Alexander von Humboldt, Bogotá, Colombia; 2 ICA—Instituto Colombiano Agropecuario, Soledad, Atlántico, Colombia; 3 Asociación GAICA, Pasto, Nariño, Colombia; 4 Grupo de Investigación en Sistemática Molecular, Universidad Nacional de Colombia, Sede Medellín, Medellín, Antioquia, Colombia; Nanjing Agricultural University, CHINA

## Abstract

Recent declines of insect populations at high rates have resulted in the need to develop a quick method to determine their diversity and to process massive data for the identification of species of highly diverse groups. A short sequence of DNA from COI is widely used for insect identification by comparing it against sequences of known species. Repositories of sequences are available online with tools that facilitate matching of the sequences of interest to a known individual. However, the performance of these tools can differ. Here we aim to assess the accuracy in identification of insect taxonomic categories from two repositories, BOLD Systems and GenBank. This was done by comparing the sequence matches between the taxonomist identification and the suggested identification from the platforms. We used 1,160 COI sequences representing eight orders of insects from Colombia. After the comparison, we reanalyzed the results from a representative subset of the data from the subfamily Scarabaeinae (Coleoptera). Overall, BOLD systems outperformed GenBank, and the performance of both engines differed by orders and other taxonomic categories (species, genus and family). Higher rates of accurate identification were obtained at family and genus levels. The accuracy was higher in BOLD for the order Coleoptera at family level, for Coleoptera and Lepidoptera at genus and species level. Other orders performed similarly in both repositories. Moreover, the Scarabaeinae subset showed that species were correctly identified only when BOLD match percentage was above 93.4% and a total of 85% of the samples were correctly assigned to a taxonomic category. These results accentuate the great potential of the identification engines to place insects accurately into their respective taxonomic categories based on DNA barcodes and highlight the reliability of BOLD Systems for insect identification in the absence of a large reference database for a highly diverse country.

## Introduction

More than a million species of plants and animals are in danger of extinction according to the latest global assessment of the intergovernmental platform on biological diversity and ecosystem services [1]. Declines in biodiversity and projections of a sixth mass extinction [2] have placed the impact of such losses on the agendas of governments all over the world [3]. Although insects constitute one of the most diverse groups on the planet, many species remain undiscovered and recent evidence suggests the decline of their populations at high rates [4, 5]. To date, the risk of extinction of 12,161 species of insects has been evaluated according to the criteria of the IUCN red list and 2,291 species are considered “threatened” around the world [6].

Colombia is known as a biodiversity hotspot in the world. Although insects are the most studied class of animals in the country [7], research involving their genetic diversity is still currently underrepresented. Countries around the world are implementing massive sampling of insects with barcode generation under the premise that barcodes will allow us to assess biodiversity at a higher speed [8]. Barcodes in taxonomic works are showing an incredibly hidden diversity of insects with descriptions of hundreds of new species facilitated by DNA, morphology, and natural history [9, 10]. As a megadiverse country, Colombia has great challenges to complete the inventory of its biodiversity and then the sustainable use of its resources. In particular, accelerating species identification using an integrative taxonomy is a priority.

Assessing biodiversity via sampling and DNA barcoding has been successfully applied in areas where coordinated sampling and barcoding facilitated the creation of extensive libraries for a particular region. An example of such collaboration is described in Morinière et al. [8] where the Bavarian State Collection of Zoology (ZSM), the Barcoding Fauna Bavarica (BFB), and the German Barcode of Life (GBOL) project generated a library of 120,000 reliably identified species in Coleoptera, Diptera, Hymenoptera, and Lepidoptera for Germany and Central Europe. A closer example in the Neotropics is Costa Rica which has been leading the Bioalfa project and within the last 15 years has generated 45,000 insect species barcodes recorded [11]. Added to this initiative are other countries across the globe such as Canada, Ecuador, Sweden, and Singapore [9, 12–16]. Moreover, the development and implementation of effective methods for species monitoring are important for assessing biodiversity, and overall health of ecosystems [8]. The species richness of insects in terrestrial ecosystems facilitates their use as bioindicators for measuring biodiversity and tracking the effects of changes in environmental conditions. The challenges faced by biodiversity declines have resulted in a need to generate new strategies for monitoring that are capable of generating vast amounts of data in a rapid and cost-effective manner. One strategy that has been gaining traction in recent years has been the implementation of DNA barcoding.

Barcode sequences are generated from a standardized region that consists of short DNA sequences (generally between ~300 to ~700 bp). The mitochondrial cytochrome c oxidase subunit I (COI) is most often used for insect barcoding [17–19]. A DNA barcoding repository is built from sampling the DNA of individuals that are retained as vouchers, from which a photograph is taken. In the case of insects, normally a leg is removed from the specimen and used to extract the DNA. In some cases, whole bodies of the samples are used when the genetic material available is limited by the size of the specimen. Although non-destructive DNA extraction methods are preferred to preserve the sample’s morphology for future reference, photographs are also suggested for facilitating the identification before this step. This approach requires the creation of a large DNA reference database where the identification of unknown specimens is achieved through the information confirmed and curated in the database. The idea behind is that unknown samples could be identified by matching their sequences to the species that are curated from the database. The Barcode of Life Data Systems BOLD (http://www.boldsystems.org) [20] is a platform specifically developed for this type of information. The BOLD platform provides the identifications match based on BINs (Barcode Index Numbers) [21] that are automatically generated through algorithms for the specimens. Although unidentified species coded in BINs can help estimating diversity, the identification of species is also possible using the Basic Local Alignment Tool (BLAST) from GenBank [22, 23]. BLAST allows matching sequences to a query sequence. The search consists of comparing nucleotide sequences hosted in the database and to provide statistical significance of these matches. The main goal from BLAST is not necessarily to provide species identification because the same principle can be used for the search of gene families; uses of identification databases are vast in biology. Scientists frequently use these two platforms to identify species from barcodes. An assessment of these two main public repositories from curated samples across taxa found a slightly better performance from GenBank vs BOLD for insects (n = 17) at species and genus level identification without statistical significance [24]. Later, a revision of this insect data found the results to be more similar than previously reported for this group [25]. Accurate identification of species and overall composition in ecosystems based on sampling is a key factor for conservation initiatives. Therefore, evaluating the performance of these platforms assigning specimens to a specific taxonomic category level has a significant impact on the advance of other fields.

Here, we analyzed a large inventory of over 1,000 insect DNA barcodes from Colombia which includes the first strepsipteran barcode for our country. We aim to compare the matches of the taxonomist identification to species, genus, and family level with the search engines of BOLD and BLAST service from GenBank in order to provide suggestions and next steps for the use of these tools. We assess the accuracy of the species identification engine from BOLD by using a subset of insects corresponding to Scarabaeinae subfamily (Coleoptera: Scarabaeidae).

## Materials and methods

### Collection locality/geography

**Fig 1:**
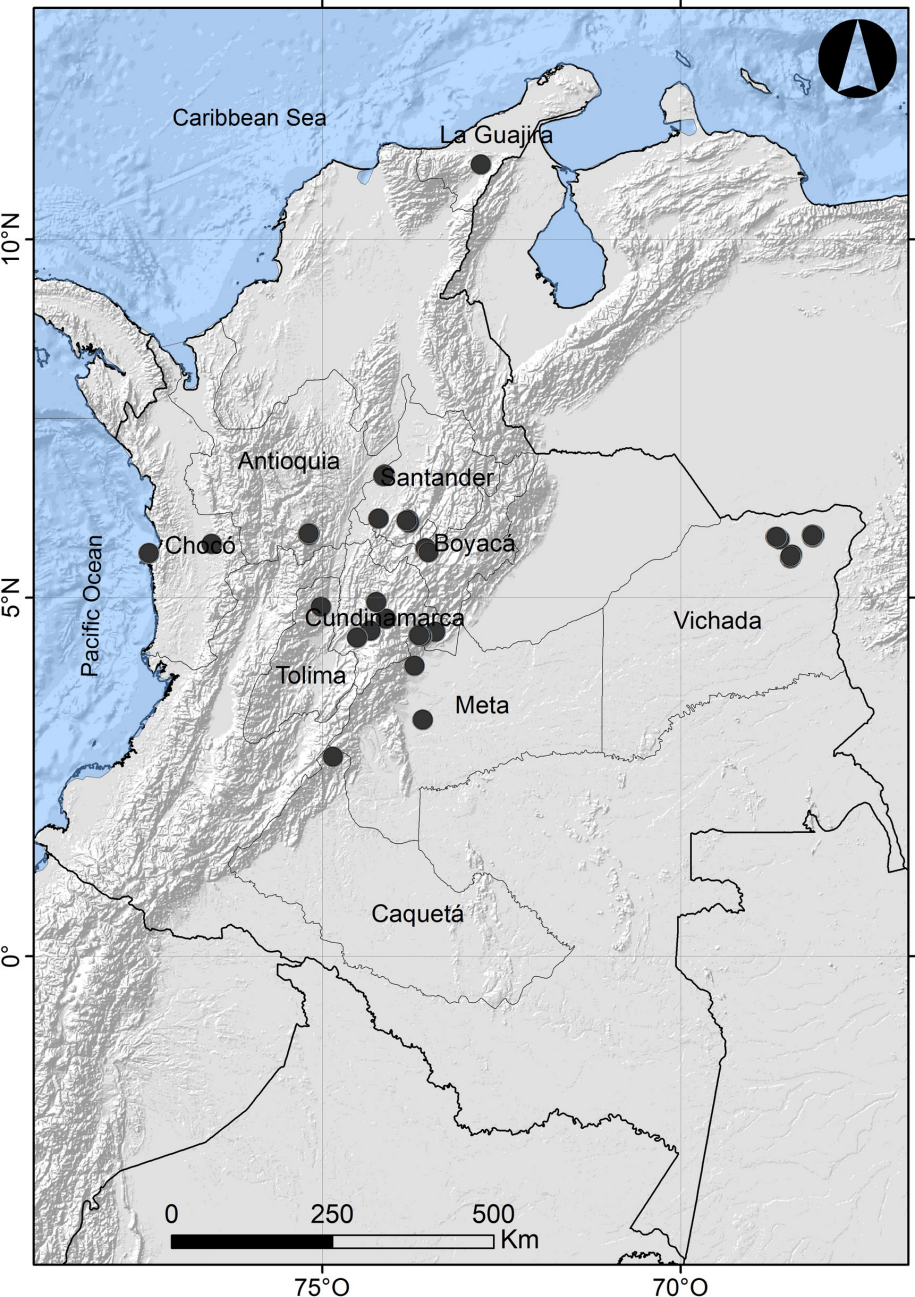
Map of Colombia showing collecting localities in black dots. Marine polygon, department boundaries, and physical labels were obtained from Natural Earth (http://www.naturalearthdata.com/). Hillshade map based on SRTM30_PLUS v8 [26].

Insect samples were collected from ten departments of Colombia through different projects (Colombia BIO, Santander BIO). The majority of the samples (81.0%) came from Antioquia, Vichada, and Santander. The spatial data of the localities were processed in ArcGIS 10.2 (License E300 04/26/2013) (Fig 1). The elevation of the samples covered a wide range with the lowest sampling occurring at 56 masl and the highest at 3,563 masl. Although samples were collected using seven different entomological collecting methods (malaise, pitfall, light trap, hand-picked, entomological net, Van Someren-Rydon trap and folding net), nine malaise traps accounted for 62.6% of the total collected material, followed by pitfall (17.2%), and light traps (8.1%). Based on the article 2.2.2.8.1.2 decree 1076 from 2015 of the environmental sector and sustainable development of Colombia, The Instituto de Investigación de Recursos Biológicos “Alexander von Humboldt” was not required to obtain a field permit when collecting specimens from the wild that are applied in non-commercial research.

### Taxa description

Our dataset included insects from the orders Coleoptera, Diptera, Hymenoptera, Lepidoptera, Hemiptera, Odonata, Psocodea and Strepsiptera, from which Coleoptera is the most represented (35%). Specimens were identified morphologically by students and by specialists from order to species level (S1 Table); additionally, for Scarabaeinae, species were identified to morphospecies level following González-Alvarado et al. [27]. Currently, few samples (7%) have been identified to lower levels from molecular data by BOLD Data Manager. Vouchers were deposited at collections with 999 samples between the Entomological and Tissue collection of the Institute Alexander von Humboldt (IAvH-E and IAvH-CT), Museo Javeriano de Historia Natural (MPUJ) at Pontificia Universidad Javeriana with 63, Museo Francisco Luis Gallego (MEFLG) at Universidad Nacional de Colombia, Sede Medellin with 55, and Colección Entomológica Universidad de Antioquia (CEUA) with the remaining 43 samples. Records of some samples are also available for search in SIB Colombia.

### DNA sampling and extraction

One leg was removed from each insect, when possible, but some samples required the use of the full body to provide enough DNA. All samples were sent to the molecular laboratory of Instituto Humboldt at CIAT, Palmira. Extraction protocols were conducted following the high throughput DNA isolation from Ivanova et al. [28]. The DNA concentration was estimated by quantification using the NanoDrop 1000 (Thermo Fisher Scientific). Amplification of cytochrome c oxidase I (COI) fragments (658 bp) took place by polymerase chain reaction (PCR) with a laboratory prepared master mixture containing 2 μL of template DNA (~10–50 ng), 1X Taq buffer ((NH4)2SO4), 200 μM of each deoxynucleoside triphosphate, 2 mM MgCl_2_, 0.2 μM of each primer, 0.4 μg/μL bovine serum albumin, and 1 U of Taq DNA polymerase. A cocktail of four primers was used: LCO1490 5’-GGTCAACAAATCATAAAGATATTGG-3’, HCO2198 5’- TAAACTTCAGGGTGACCAAAAAATCA-3’ [29], Lep-F1 5’- ATTCAACCAATCATAAAGATATTGG-3’ and Lep-R1 5’- TAAACTTCTGGATGTCCAAAAAATCA-3’ [30]. Primers were selected following Canadian Centre for DNA Barcoding (CCDB) suggested primers for taxonomic groups. The PCR cycling conditions consisted of a first cycle of denaturation at 94°C for 3 min; followed by 5 cycles of 94°C for 30 s, 45°C for 40 s, and 72°C for 1 min; then 35 cycles of 94°C for 30 s, 51°C for 40 s, and 72°C for 1 min; and a final extension cycle at 72°C for 5 min. PCR products were visualized by electrophoresis on 1.5% agarose gels stained with SYBR Safe (Thermo Fisher Scientific) and using 1X TBE. The ExoSAP-IT protocol was used to clean PCR products, after which Sanger sequencing of amplicons were carried out at The Universidad Nacional de Colombia and the University of the Andes. Sequences were assembled and edited in Geneious 10.0.9 [31]. All files were uploaded in BOLD systems and are found in the dataset DS-CBIH2020; Dataset available at: doi.org/10.5883/DS-CBIH2020.

### Data analysis

#### BOLD Batch ID engine

To identify samples to species level, we ran BOLD’s Identification Engine in datasets created for insect orders in July 2020. The identification engine allows the user to select the search within the COI Species Database or COI Full Database. We selected COI Species Database which will match the sequence against an identified species as opposed to individuals without identification to species level. We kept filters as default for a minimum overlap of 300 bp and sequence length ≥ 100 bp. We ran the analysis with 97% similarity, a percentage that accounts for within species variation and error of the sequence [8]. Under the criteria selected of 97% numerous species ID were not obtained. We decided to decrease the criteria and include matches using the 80% similarity filter due to the lack of matches at 97%. The 80% similarity is the minimum filter allowed in BOLD. A match was accepted as correct when the identification by the taxonomist was in agreement by the suggested identification by BOLD. The ID search engine provides a list of maximum 100 closest specimens, and we selected the first suggestion from the list that has the highest percentage match and base pair overlap. We included an alternative match when the suggestion was done towards a sequence from our project and ran statistical tests separately to prevent circular analysis of results. Matches were checked at species, genus, and family level.

#### BLAST

We used BLAST from GenBank in Geneious 10.0.9 by default to look for matches of the sequences. The parameters included were the database search from Nucleotides collection, program set as the MegaBlast, results displayed as a Hit table, and retrieve matching region with maximum hits of 100. The search was set to provide a list of 100 closest nucleotide sequences, from which we selected the first suggestion that obtained the highest Bit-score or Max score. Most of the times, the E value was 0 (or closest to zero) and the query cover was 100%. Matches were checked at species, genus and family level.

The identification analyses in both approaches (BOLD and BLAST) followed the assumption that species included on the databases are correctly identified; however, this approach might not be accurate in cases where the most similar species were wrongly identified.

#### Dung beetles (Scarabaeinae) case study

The Scarabaeinae subfamily was selected due to the high representation of this group in the samples of Coleoptera (42%) and from the total insect samples (15%). In order to evaluate the accuracy of species identification suggested by BOLD, we consulted taxonomists with expertise in the identification of the Scarabaeidae family. They were asked to reanalyzed the results and mention if they agree with the species and genus identification suggested by BOLD and to explain why, as follows: Correctly identified genus, correctly identified species, probable correctly identified species, incorrect identification (Table 1). When available, specimens deposited at the IAVH-E collection and photographs uploaded to BOLD were checked for specimens to corroborate the taxonomy, and field notes from the collectors.

**Table 1:** List of categories used by the taxonomist to define the identification results suggested by BOLD with the Scarabaeinae subfamily.

Category	Definition
Correctly identified genus	Agreement between the taxonomist and BOLD at the genus level
Correctly identified species	Agreement between the taxonomist and BOLD at the species level
Probable correctly identified species	Agreement between the taxonomist morphospecies and the species suggested by BOLD (the genus matched and probable species)
Incorrect identification	Disagreement between the taxonomist and BOLD (the genus or species did not match)

#### Statistical analysis

Chi-square analyses were performed between match results for BOLD and GenBank at the studied taxonomic levels (species, genera and family) to evaluate if there was a significant difference between the platforms performance. An additional series of Chi-square analyses were performed to identify differences in the platforms by each order of insect at the species, genus, and family level. We adjusted our significance threshold at each taxonomic level based on the number of orders compared using the Bonferroni Correction. Flagged records were removed from the analysis.

The non-parametric Kruskal-Wallis test in PAST, Version 4.05 [32] was used to determine any significant differences of medians for BOLD % match of the correct Scarabaeinae categories against incorrect identification. Assumptions for an ANOVA were not fulfilled. Dunn’s post hoc was carried out to identify differences between categories.

## Results

### Description of the data

**Fig 2:**
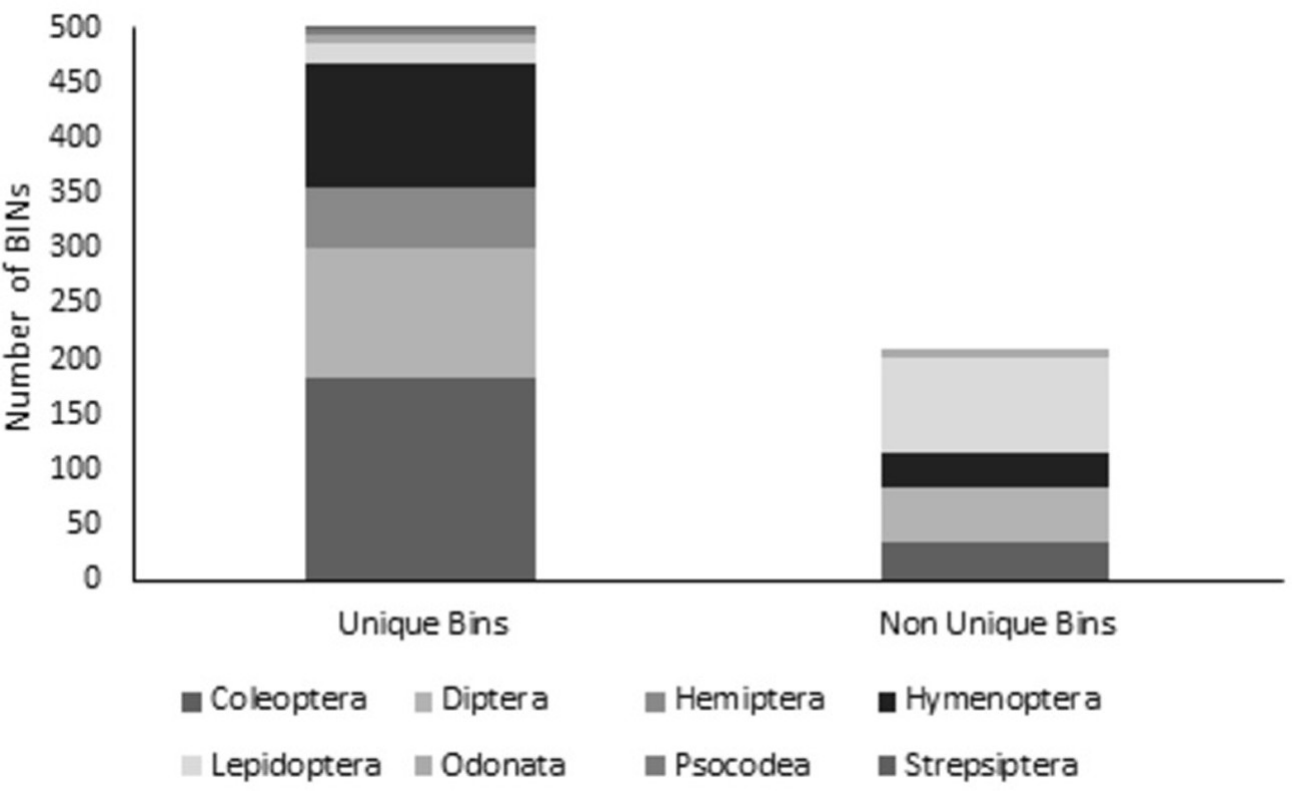
Summary of Barcode Index Numbers (BINS) obtained by orders of insects. A total of 500 BINs were identified as unique and 208 BINs as non-unique in the platform BOLD Systems.

A total of 1,160 COI sequences of insects from Colombia were generated since 2019 (S1 Table). From this set, 1,088 sequences were barcode compliant and placed in 708 BINS (Table 2). Of these 708 BINS, 500 were identified as unique (Fig 2). The most frequent BINS were all in Coleoptera, BOLD:ADN3981 *Paulosawaya* sp. (11 specimens), BOLD:ADO6609 *Paulosawaya ursina* (Blanchard, 1850) (10 specimens), and BOLD:ADJ9394 Ptilodactylidae (9 specimens). From the specimens with barcode compliant 33 were flagged as problematic records in most instances due to suspected contamination, specimen mix-up or misidentification.

**Table 2:** Summary of specimens and barcodes data by orders after removal of flagged records.

Order	Specimens #	Flagged record	Family count	Specimen count IDed Family	Unknown	Genera count	Specimen count IDed Genus	Species count	Specimen count IDed Species	Barcode compliant	Species count	BINS	Unique Bins	Non-Unique Bins
Coleoptera	411	14	25	397	0	57	271	63	117	369	57	218	184	34
Diptera	277	14	24	257	6	4	22	0	N/A	257	0	165	116	49
Hemiptera	82	4	11	78	0	0	N/A	0	N/A	68	0	58	56	2
Hymenoptera	201	2	10	199	0	16	47	2	3	176	2	142	112	30
Lepidoptera	166	0	8	161	5	57	154	79	128	163	73	104	18	86
Odonata	17	0	6	17	0	14	17	13	15	16	13	15	8	7
Psocodea	5	0	4	5	0	0	N/A	N/A	N/A	5	N/A	5	5	0
Strepsiptera	1	0	1	1	0	1	1	0	N/A	1	0	1	1	0
**Total**	**1160**	**34**	**89**	**1115**	**11**	**149**	**512**	**157**	**263**	**1055**	**145**	**708**	**500**	**208**

### Performance of BOLD and BLAST (GenBank) identification based on taxonomist match

**Fig 3:**
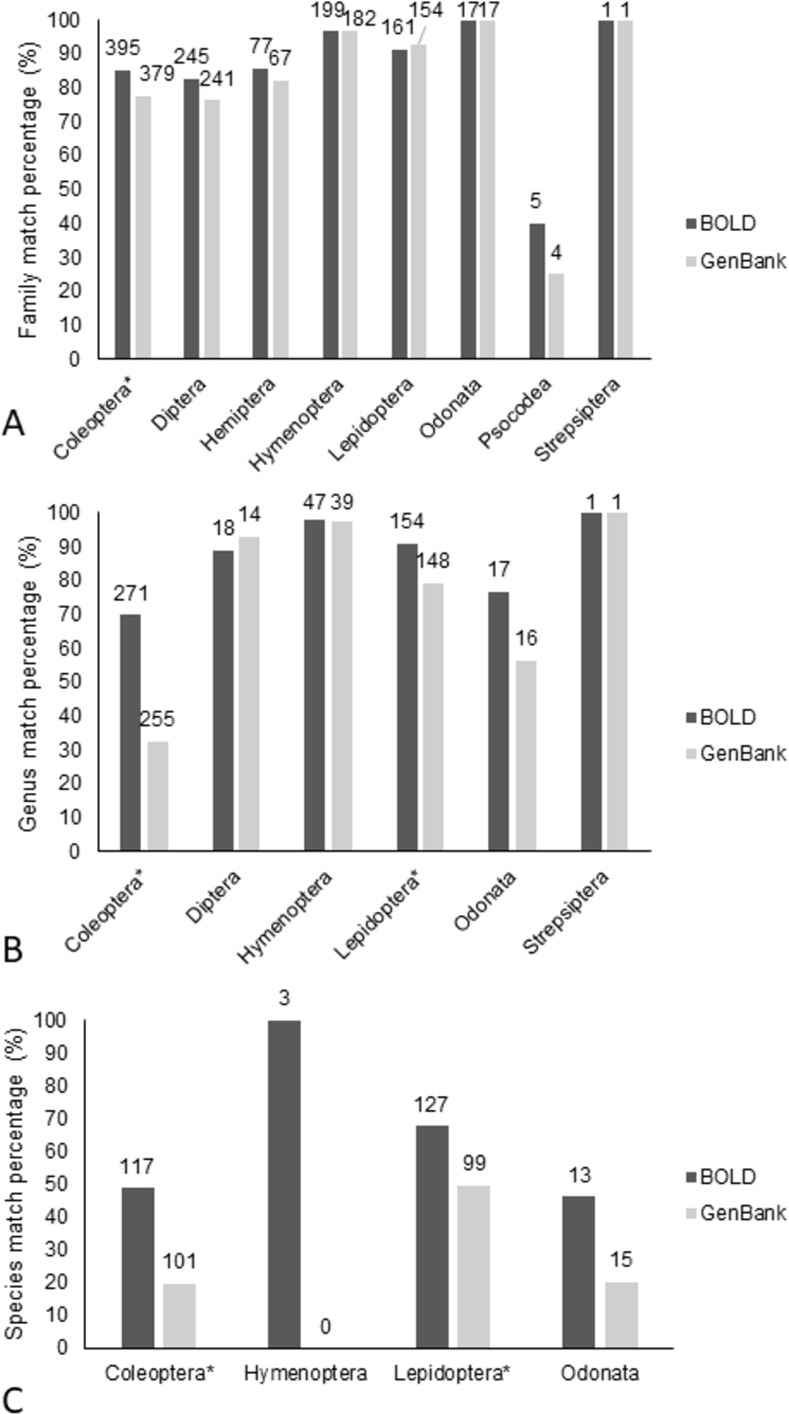
Comparison by order of performance of BOLD Systems and BLAST for identification of specimens in percentages. Correct matches for BOLD Batch ID engine at 80% are shown in dark gray. Correct matches from MegaBlast search by default (GenBank) are shown in light gray. *Is next to orders where Chi-square analyzed with Bonferroni correction was significant. The sample size is displayed on top of each column. A) by families. B) by genera C) by species.

Identifications provided by BOLD engine and BLAST are available in S2 Table as well as the matches in agreement with the taxonomist. The performance of BOLD vs BLAST for identification of specimens was significantly greater with BOLD for the pooled comparison of all species (x2 = 29.5, df = 1, P < 0.001), genera (x2 = 67.7, df = 1, P < 0.001), and families (x2 = 8.3, df = 1, P = 0.004). Comparisons of the performance of BOLD and BLAST by orders at the different taxonomic levels revealed significantly more matches with BOLD (Fig 3), at the family level (Fig 3A) in Coleoptera (x2 = 7.7, df = 1, P = 0.006), at genus level (Fig 3B) in Coleoptera (x2 = 72.8, df = 1, P < 0.001) and Lepidoptera (x2 = 8.4, df = 1, P = 0.004) and at the species level (Fig 3C) in Coleoptera (x2 = 19.8, df = 1, P < 0.001) and Lepidoptera (x2 = 7.7, df = 1, P = 0.006). No differences were found for the other orders tested at the different taxonomic categories. Same results were found when analyses were performed with matches to data from our project, the only comparison that changed was at the genus level where no significant differences were found for Lepidoptera (S3 Table).

### Case of study Scarabaeinae subfamily (Coleoptera: Scarabaeidae)

A total of 172 specimens were analyzed. The samples in the subfamily Scarabaeinae were assigned by the taxonomist to 16 genera and 27 species (S1 Table).

**Fig 4:**
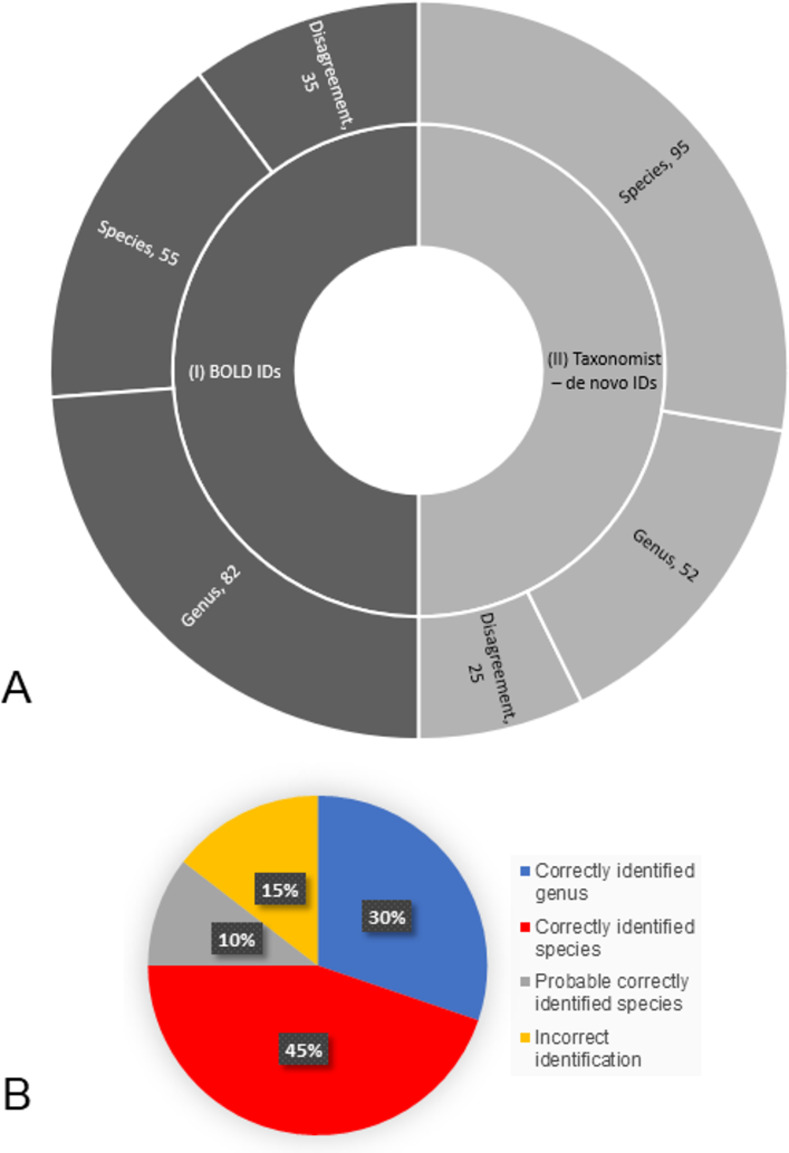
Results of the reanalysis performed by the taxonomist in the subfamily of dung beetles Scarabaeinae (Coleoptera: Scarabaeidae). A) Comparison between the identification (ID) matches before (I, BOLD IDs) and after (II, taxonomist–*de novo* IDs) reanalyzing the data. B) Percentage of the categories assigned by the taxonomist to the identifications suggested with BOLD’s search engine at 80%.

BOLD engine with the search at 80% provided identifications for all 172 specimens analyzed (Fig 4A and S2 Table). A match in agreement with the taxonomist was found for 82 (80%) specimens out of 103 at the genus level. For species, the search resulted in 55 (78%) specimens out of 69 in total. Based on this information, these records were classified as 137 (80%) in agreement with the taxonomist and 35 (20%) in disagreement (Table 3 and Fig 4A).

**Table 3:** Summary of matches between the taxonomist and BOLD identifications for the subfamily of dung beetles Scarabaeinae. The first section (I) corresponds to the initial matches performed in agreement with the taxonomist after the search engine in BOLD at 80%. The second section (II) in grey includes the categories used by the taxonomist to reevaluate the identifications provided by BOLD in (I) and the final matches in agreement.

				Matches BOLD & Taxonomist	Count	Total	%	Difference % (disagreement with taxonomist)
(I) BOLD search engine identifications				Genus	82	103	80	
				Species	55	69	78	
				**Sum agreement**	**137**	172	**80**	20
	**Categories**	**Count**	**%**					
(II) Taxonomist corroborated identifications–*de novo*	Correctly identified genus	52	30	Genus	52	73	71	
	Correctly identified species	77	45	Species	95	99	96	
	Probable correctly identified species	18	10					
	Incorrect identification	25	15	**Sum agreement**	**147**	172	**85**	15

Then, we corroborated *de novo* the identification for these 172 records of dung beetle with a taxonomist (Fig 4B). The *de novo* specimens were assigned to the same 16 genera and 27 species (70 specimens), in addition to 31 morphospecies (genus and code "H", 97 specimens) (Tables 4 and S4). The new review resulted in 95 records (55%) correctly matched the taxonomist species identification or was deemed likely to be the species (Correctly identified species and probable correctly identified species categories). At the genus level, 52 records (30%) correctly matched the taxonomist genus identification but either were not accurately matched with the species or the species identification was not provided (Correctly identified genus category). There were 25 records (15%) at the genus level which did not match the identification of the taxonomist and arguments against BOLD identification were provided by the taxonomist (Incorrect identification category) (S4 Table). Therefore, the records previously classified as 137 (80%) increased to 147 (85%) in agreement with the taxonomist and were reduced from 35 (20%) to 25 (15%) in disagreement based on the BOLD search engine at 80% (Table 3 and Fig 4A).

**Table 4:** List of Scarabaeinae species identified by BOLD in agreement with the taxonomist’s *de novo* identification. Morphospecies with genus and code "H" are included [27]. Only categories for correct species and probably correct species are presented here.

Process ID	BIN	Current ID	% Match	Overlap (bp)	Taxonomist’s *de novo* ID	Match Species BOLD	Match BIN	Match notes
CBIHM686-18	BOLD:ACW4694	*Ateuchus*	93.4	568	*Ateuchus aeneomicans*	*Ateuchus aeneomicans*	BOLD:AAW8107	Correct species
CBIHI151-18	BOLD:ADL3091	*Canthon juvencus*	99.85	567	*Canthon juvencus*	*Canthon juvencus*	BOLD:ADL3091	Correct species
CBIHM706-18	BOLD:ADL3091	*Canthon juvencus*	99.85	567	*Canthon juvencus*	*Canthon juvencus*	BOLD:ADL3091	Correct species
CBIHM705-18	BOLD:ADL3091	*Canthon juvencus*	99.69	566	*Canthon juvencus*	*Canthon juvencus*	BOLD:ADL3091	Correct species
CBIHM633-18		*Canthon*	98.2	563	*Sylvicanthon aequinoctialis* [33]	*Canthon aequinoctialis*	BOLD:ABA2649	Correct species
CBIHM627-18	BOLD:ABA2649	*Canthon*	98.01	561	*Sylvicanthon aequinoctialis*	*Canthon aequinoctialis*	BOLD:ABA2649	Correct species
CBIHM635-18	BOLD:ABA2649	*Canthon*	97.55	570	*Sylvicanthon aequinoctialis*	*Canthon aequinoctialis*	BOLD:ABA2649	Correct species
CBIHM636-18	BOLD:ABA2649	*Canthon*	97.55	570	*Sylvicanthon aequinoctialis*	*Canthon aequinoctialis*	BOLD:ABA2649	Correct species
CBIHM632-18	BOLD:ABA2649	*Canthon*	97.25	570	*Sylvicanthon aequinoctialis*	*Canthon aequinoctialis*	BOLD:ABA2649	Correct species
CBIHM631-18	BOLD:ABA2647	*Canthon*	100	570	*Sylvicanthon aequinoctialis*	*Canthon aequinoctialisASolis1*	BOLD:ABA2647	Correct species
CBIHM637-18	BOLD:ABA2647	*Canthon*	100	570	*Sylvicanthon aequinoctialis*	*Canthon aequinoctialisASolis1*	BOLD:ABA2647	Correct species
CBIHM638-18	BOLD:ABA2647	*Canthon*	100	570	*Sylvicanthon aequinoctialis*	*Canthon aequinoctialisASolis1*	BOLD:ABA2647	Correct species
CBIHM634-18	BOLD:ABA2647	*Canthon*	99.85	570	*Sylvicanthon aequinoctialis*	*Canthon aequinoctialisASolis1*	BOLD:ABA2647	Correct species
CBIHM629-18	BOLD:ABA2649	*Canthon*	98.17	570	*Sylvicanthon aequinoctialis*	*Canthon aequinoctialisASolis3*	BOLD:ABA2649	Correct species
CBIHM628-18	BOLD:ABA2649	*Canthon*	98.08	570	*Sylvicanthon aequinoctialis*	*Canthon aequinoctialisASolis3*	BOLD:ABA2649	Correct species
CBIHM639-18	BOLD:ABA2649	*Canthon*	97.86	570	*Sylvicanthon aequinoctialis*	*Canthon aequinoctialisASolis3*	BOLD:ABA2649	Correct species
CBIHM747-18	BOLD:ACW3356	*Canthon subhyalinus*	100	570	*Canthon subhyalinus*	*Canthon subhyalinus*	BOLD:ACW3356	Correct species
CBIHM673-18	BOLD:ACW3356	*Canthon subhyalinus*	100	570	*Canthon subhyalinus*	*Canthon subhyalinus*	BOLD:ACW3356	Correct species
CBIHM674-18	BOLD:ACW3356	*Canthon subhyalinus*	100	570	*Canthon subhyalinus*	*Canthon subhyalinus*	BOLD:ACW3356	Correct species
CBIHM727-18	BOLD:ACW3356	*Canthon subhyalinus*	100	570	*Canthon subhyalinus*	*Canthon subhyalinus*	BOLD:ACW3356	Correct species
CBIHM744-18	BOLD:ACW3356	*Canthon subhyalinus*	100	567	*Canthon subhyalinus*	*Canthon subhyalinus*	BOLD:ACW3356	Correct species
CBIHM718-18	BOLD:ACW3356	*Canthon subhyalinus*	99.24	570	*Canthon subhyalinus*	*Canthon subhyalinus*	BOLD:ACW3356	Correct species
CBIHM716-18	BOLD:ACW3356	*Canthon subhyalinus*	98.93	570	*Canthon subhyalinus*	*Canthon subhyalinus*	BOLD:ACW3356	Correct species
CBIHM717-18	BOLD:ACW3356	*Canthon subhyalinus*	98.93	570	*Canthon subhyalinus*	*Canthon subhyalinus*	BOLD:ACW3356	Correct species
CBIHM741-18	BOLD:ACW3356	*Canthon*	100	570	*Canthon subhyalinus*	*Canthon subhyalinus*	BOLD:ACW3356	Correct species
CBIHI156-18	BOLD:ADO2303	*Canthon triangularis*	100	570	*Canthon triangularis*	*Canthon triangularis*	BOLD:ADO2303	Correct species
CBIHI157-18	BOLD:ADO2303	*Canthon triangularis*	100	570	*Canthon triangularis*	*Canthon triangularis*	BOLD:ADO2303	Correct species
CBIHM659-18	BOLD:ADL4806	*Coprophanaeus corythus*	97.24	540	*Coprophanaeus corythus*	*Coprophanaeus corythus*	BOLD:ACW3300	Correct species
CBIHM687-18	BOLD:ADL4607	*Coprophanaeus gamezi*	94.65	570	*Coprophanaeus gamezi*	*Coprophanaeus gamezi*	BOLD:ACW3623	Correct species
CBIHI019-17	BOLD:ADJ7028	*Deltochilum mexicanum*	95.26	570	*Deltochilum mexicanum*	*Deltochilum mexicanum*	BOLD:AAP9113	Correct species
CBIHI159-18	BOLD:ACW3574	*Dichotomius*	95.61	570	*Dichotomius agenor*	*Dichotomius agenor*	BOLD:ABA2814	Correct species
CBIHM648-18	BOLD:ACW3574	*Dichotomius*	95.61	570	*Dichotomius agenor*	*Dichotomius agenor*	BOLD:ABA2814	Correct species
CBIHM667-18	BOLD:ACW3574	*Dichotomius*	95.36	570	*Dichotomius agenor*	*Dichotomius agenor*	BOLD:ABA2814	Correct species
CBIHM666-18	BOLD:ACW3574	*Dichotomius*	95.28	570	*Dichotomius agenor*	*Dichotomius agenor*	BOLD:ABA2814	Correct species
CBIHM646-18	BOLD:ACW3574	*Dichotomius*	95.23	570	*Dichotomius agenor*	*Dichotomius agenor*	BOLD:ABA2814	Correct species
CBIHM647-18	BOLD:ACW3574	*Dichotomius*	95.21	567	*Dichotomius agenor*	*Dichotomius agenor*	BOLD:ABA2814	Correct species
CBIHM645-18	BOLD:ACW3574	*Dichotomius*	95.12	570	*Dichotomius agenor*	*Dichotomius agenor*	BOLD:ABA2814	Correct species
CBIHM665-18	BOLD:ACW3574	*Dichotomius*	95.12	570	*Dichotomius agenor*	*Dichotomius agenor*	BOLD:ABA2814	Correct species
CBIHI144-18	BOLD:AAC9021	*Digitonthophagus gazella*	100	570	*Digitonthophagus gazella*	*Digitonthophagus gazella*	BOLD:AAC9021	Correct species
CBIHM640-18	BOLD:ADL2649	*Eurysternus caribaeus*	96.48	570	*Eurysternus caribaeus*	*Eurysternus caribaeus*	BOLD:ABA7207	Correct species
CBIHI145-18	BOLD:ACW4232	*Eurysternus caribaeus*	100	570	*Eurysternus caribaeus*	*Eurysternus caribaeus*	BOLD:ACW4232	Correct species
CBIHI146-18	BOLD:ACW4232	*Eurysternus caribaeus*	100	570	*Eurysternus caribaeus*	*Eurysternus caribaeus*	BOLD:ACW4232	Correct species
CBIHI147-18	BOLD:ACW4232	*Eurysternus caribaeus*	100	570	*Eurysternus caribaeus*	*Eurysternus caribaeus*	BOLD:ACW4232	Correct species
CBIHI023-17	BOLD:ADJ7677	*Eurysternus foedus*	95.57	570	*Eurysternus foedus*	*Eurysternus foedus*	BOLD:ABU6799	Correct species
CBIHI030-17	BOLD:AAX0271	*Onthophagus acuminatus*	100	570	*Onthophagus acuminatus*	*Onthophagus acuminatusAS1*	BOLD:AAX0271	Correct species
CBIHM653-18	BOLD:ADL2546	*Eurysternus mexicanus*	100	570	*Eurysternus mexicanus*	*Eurysternus mexicanus*	BOLD:ADL2546	Correct species
CBIHM654-18	BOLD:ADL2546	*Eurysternus mexicanus*	100	570	*Eurysternus mexicanus*	*Eurysternus mexicanus*	BOLD:ADL2546	Correct species
CBIHM655-18	BOLD:ADL2546	*Eurysternus mexicanus*	100	570	*Eurysternus mexicanus*	*Eurysternus mexicanus*	BOLD:ADL2546	Correct species
CBIHM656-18	BOLD:ADL2546	*Eurysternus mexicanus*	100	570	*Eurysternus mexicanus*	*Eurysternus mexicanus*	BOLD:ADL2546	Correct species
CBIHM660-18	BOLD:ADL2546	*Eurysternus mexicanus*	100	570	*Eurysternus mexicanus*	*Eurysternus mexicanus*	BOLD:ADL2546	Correct species
CBIHM661-18	BOLD:ADL2546	*Eurysternus mexicanus*	100	570	*Eurysternus mexicanus*	*Eurysternus mexicanus*	BOLD:ADL2546	Correct species
CBIHM662-18	BOLD:ADL2546	*Eurysternus mexicanus*	100	570	*Eurysternus mexicanus*	*Eurysternus mexicanus*	BOLD:ADL2546	Correct species
CBIHM657-18	BOLD:ADL2546	*Eurysternus mexicanus*	99.83	570	*Eurysternus mexicanus*	*Eurysternus mexicanus*	BOLD:ADL2546	Correct species
CBIHM698-18	BOLD:ADL4924	*Malagoniella astyanax*	94.34	570	*Malagoniella astyanax*	*Malagoniella astyanax*	BOLD:ABA3615	Correct species
CBIHM701-18	BOLD:ACW3925	*Ontherus appendiculatus*	99	558	*Ontherus appendiculatus*	*Ontherus appendiculatus*	BOLD:ACW3925	Correct species
CBIHM703-18	BOLD:ACW3925	*Ontherus appendiculatus*	99	570	*Ontherus appendiculatus*	*Ontherus appendiculatus*	BOLD:ACW3925	Correct species
CBIHM699-18	BOLD:ACW3925	*Ontherus appendiculatus*	98.95	570	*Ontherus appendiculatus*	*Ontherus appendiculatus*	BOLD:ACW3925	Correct species
CBIHM702-18	BOLD:ACW3925	*Ontherus appendiculatus*	98.5	558	*Ontherus appendiculatus*	*Ontherus appendiculatus*	BOLD:ACW3925	Correct species
CBIHI160-18		*Onthophagus curvicornis*	99.82	552	*Onthophagus curvicornis*	*Onthophagus curvicornis*	BOLD:ADM9496	Correct species
CBIHI142-18	BOLD:ADM9497	*Onthophagus curvicornis*	98.17	570	*Onthophagus curvicornis*	*Onthophagus curvicornis*	BOLD:ADM9497	Correct species
CBIHI161-18	BOLD:ADM9497	*Onthophagus curvicornis*	98.17	570	*Onthophagus curvicornis*	*Onthophagus curvicornis*	BOLD:ADM9497	Correct species
CBIHI162-18	BOLD:ADM9496	*Onthophagus curvicornis*	99.82	567	*Onthophagus curvicornis*	*Onthophagus curvicornis*		Correct species
CBIHM719-18	BOLD:ADL5334	*Onthophagus lebasi*	99.84	569	*Onthophagus lebasi*	*Onthophagus lebasi*	BOLD:ADL5334	Correct species
CBIHM735-18	BOLD:ADL5334	*Onthophagus lebasi*	99.84	567	*Onthophagus lebasi*	*Onthophagus lebasi*	BOLD:ADL5334	Correct species
CBIHM726-18	BOLD:ADL5334	*Onthophagus lebasi*	99.35	567	*Onthophagus lebasi*	*Onthophagus lebasi*	BOLD:ADL5334	Correct species
CBIHM721-18	BOLD:ADL5334	*Onthophagus lebasi*	98.61	566	*Onthophagus lebasi*	*Onthophagus lebasi*	BOLD:ADL5334	Correct species
CBIHI152-18	BOLD:ADM9873	*Phanaeus bispinus*	96.94	570	*Phanaeus bispinus*	*Phanaeus bispinus*	BOLD:ACW4591	Correct species
CBIHI153-18	BOLD:ADN3451	*Phanaeus haroldi*	98.62	570	*Phanaeus haroldi*	*Phanaeus haroldi*	BOLD:ADN3451	Correct species
CBIHI154-18	BOLD:ADN3451	*Phanaeus haroldi*	98.62	570	*Phanaeus haroldi*	*Phanaeus haroldi*	BOLD:ADN3451	Correct species
CBIHM712-18	BOLD:ACW3786	*Sulcophanaeus leander*	99.83	570	*Sulcophanaeus leander*	*Sulcophanaeus leander*	BOLD:ACW3786	Correct species
CBIHM713-18	BOLD:ACW3786	*Sulcophanaeus leander*	99.68	570	*Sulcophanaeus leander*	*Sulcophanaeus leander*	BOLD:ACW3786	Correct species
CBIHM711-18	BOLD:ACW3786	*Sulcophanaeus leander*	99.66	570	*Sulcophanaeus leander*	*Sulcophanaeus leander*	BOLD:ACW3786	Correct species
CBIHM729-18	BOLD:ADL4592	*Uroxys micros*	100	504	*Uroxys micros*	*Uroxys micros*	BOLD:ADL4592	Correct species
CBIHM731-18	BOLD:ADL4592	*Uroxys micros*	100	501	*Uroxys micros*	*Uroxys micros*	BOLD:ADL4592	Correct species
CBIHM740-18	BOLD:ADL4592	*Uroxys micros*	100	504	*Uroxys micros*	*Uroxys micros*	BOLD:ADL4592	Correct species
CBIHM746-18	BOLD:ADL4592	*Uroxys micros*	99.83	513	*Uroxys micros*	*Uroxys micros*	BOLD:ADL4592	Correct species
CBIHM739-18	BOLD:ADL4592	*Uroxys micros*	99.16	494	*Uroxys micros*	*Uroxys micros*	BOLD:ADL4592	Correct species
CBIHI025-17	BOLD:ADK7115	*Canthidium*	95.39	567	*Canthidium* sp. 21H	*Canthidium centrale*	BOLD:ABU9359	Probably correct species
CBIHM650-18	BOLD:ACW2842	*Canthon*	94.97	567	*Canthon* sp. 06H	*Canthon cyanellus*	BOLD:ADN2169	Probably correct species
CBIHM651-18	BOLD:ACW2842	*Canthon*	94.97	567	*Canthon* sp. 06H	*Canthon cyanellus*	BOLD:ADN2169	Probably correct species
CBIHM663-18	BOLD:ACW2842	*Canthon*	94.9	570	*Canthon* sp. 06H	*Canthon cyanellus*	BOLD:ADN2169	Probably correct species
CBIHM664-18	BOLD:ACW2842	*Canthon*	94.9	570	*Canthon* sp. 06H	*Canthon cyanellus*	BOLD:ADN2169	Probably correct species
CBIHM649-18	BOLD:ACW2842	*Canthon*	94.85	567	*Canthon* sp. 06H	*Canthon cyanellus*	BOLD:ADN2169	Probably correct species
CBIHM742-18	BOLD:ACW4541	*Canthon*	96.83	570	*Canthon* sp. 09H	*Canthon caelius*	BOLD:ABU7368	Probably correct species
CBIHM668-18	BOLD:ACW4541	*Canthon*	96.62	567	*Canthon* sp. 09H	*Canthon caelius*	BOLD:ABU7368	Probably correct species
CBIHM669-18	BOLD:ACW4541	*Canthon*	96.62	567	*Canthon* sp. 09H	*Canthon caelius*	BOLD:ABU7368	Probably correct species
CBIHM670-18	BOLD:ACW4541	*Canthon*	96.62	567	*Canthon* sp. 09H	*Canthon caelius*	BOLD:ABU7368	Probably correct species
CBIHM730-18	BOLD:ACW4541	*Canthon*	96.62	567	*Canthon* sp. 09H	*Canthon caelius*	BOLD:ABU7368	Probably correct species
CBIHM732-18	BOLD:ACW4541	*Canthon*	96.6	570	*Canthon* sp. 09H	*Canthon caelius*	BOLD:ABU7368	Probably correct species
CBIHM820-20		*Canthon*	95.11	408	*Canthon* sp. 09H	*Canthon caelius*	BOLD:ABU7368	Probably correct species
CBIHM683-18	BOLD:ADL3950	*Canthon*	95.01	570	*Canthon* sp. 23H	*Canthon bispinus*	BOLD:ADR8659	Probably correct species
CBIHM684-18	BOLD:ADL3950	*Canthon*	95.01	570	*Canthon* sp. 23H	*Canthon bispinus*	BOLD:ADR8659	Probably correct species
CBIHI148-18	BOLD:AAX0252	*Onthophagus*	98.04	570	*Onthophagus* sp. 07H	*Onthophagus haematopus*	BOLD:AAX0252	Probably correct species
CBIHI150-18	BOLD:AAX0252	*Onthophagus*	98.04	570	*Onthophagus* sp. 07H	*Onthophagus haematopus*	BOLD:AAX0252	Probably correct species
CBIHI149-18	BOLD:AAX0252	*Onthophagus*	97.88	570	*Onthophagus* sp. 07H	*Onthophagus haematopus*	BOLD:AAX0252	Probably correct species

**Fig 5:**
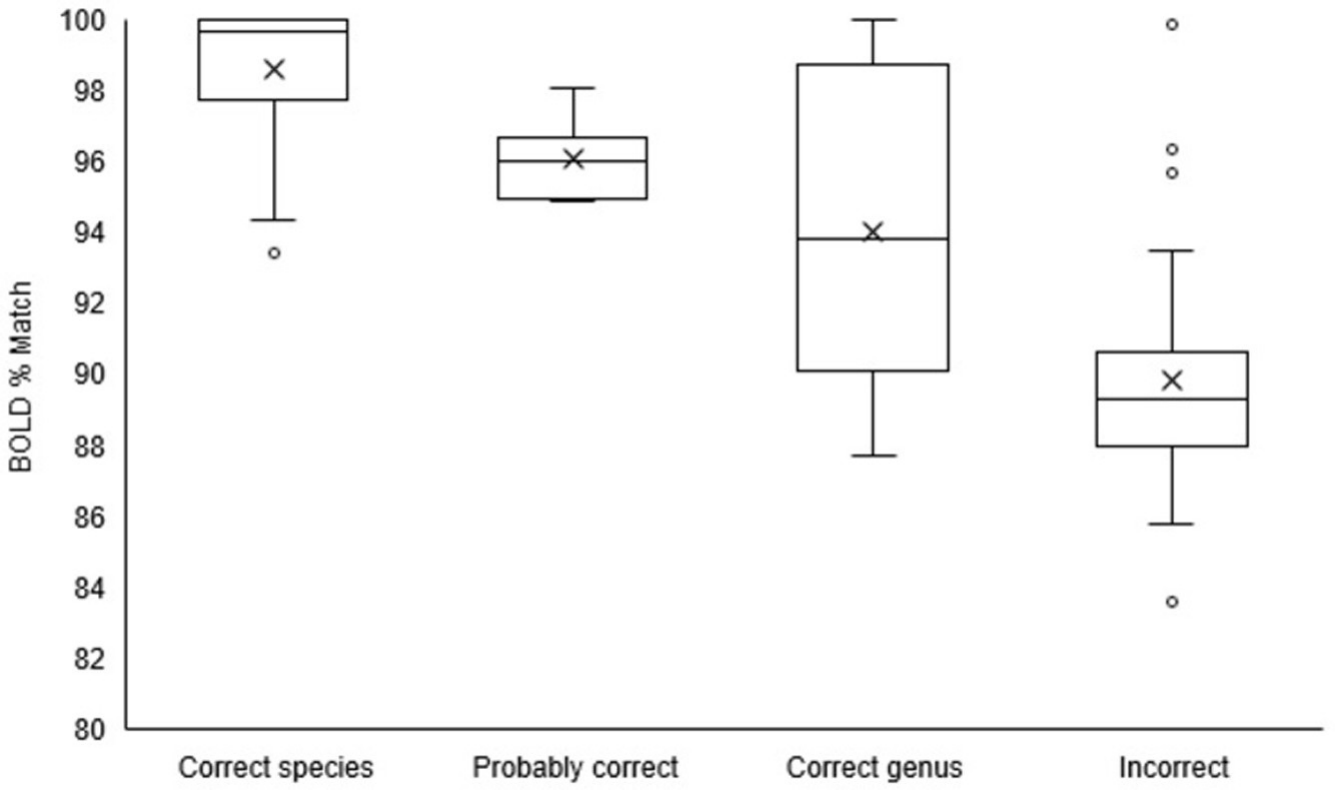
Boxplot of Scarabaeinae case of study showing the range of BOLD percentage matches for the categories: Correctly Identified species, probable correctly identified species, correctly identified genus, and incorrect identification. The median is represented by a line, the mean is represented by an X markers and outliers are the white dots.

Correctly identified species had a percentage match range in BOLD from 93.4 to 100 (Fig 5). Probable correctly identified species ranged from 94.85 to 98.04. Correctly identified genus range was between 87.7 and 100%. Incorrect identification percentages range from 83.59 to 99.83. The categories were significantly different (Kruskal-Wallis H = 73.4, P < 0.001). The correctly identified species (P < 0.001), probable correctly identified species (P < 0.05) and correctly identified genus (P < 0.05) categories were different from the incorrect identifications.

## Discussion

In our study, BOLD systems outperformed BLAST, providing more accurate insect identifications. This overall pattern was found for all taxonomic levels evaluated (species, genus and family). Although differences were found between taxa, BOLD consistently provided higher identifications for the order Coleoptera at all taxonomic levels and for Lepidoptera at genus and species level. For instance, Hymenoptera and Odonata performed similarly for both repositories at all taxonomic levels, alike most of the other orders at the family level when analyzed separately. These results are partially in disagreement with Meiklejohn et al. [24] which reported no significant differences between BOLD and GenBank for insect samples identification at genus and species level. A further review of their data showed that correct identifications from both platforms were closer than estimated previously [25]. Although we report different results in the performance of BOLD compared to GenBank in this study, a key factor to consider is the magnitude of insect data involved. Meiklejohn et al. [24], who studied 17 specimens in 12 orders at genus and species level using COI whereas we included 1,160 specimens in eight orders at family, genus and species level. BOLD Systems is a platform developed with the intention of providing species identification and in that sense does not perform equally to GenBank, but both are the main public repositories often used to provide identifications. Often BOLD is considered to contain data more curated, but GenBank data quality is good overall [25, 34]. Differences in identifications of taxa can be considered due to differential taxonomic curation of the databases and the lack of barcodes of reference from Colombia. However, we could not identify a clear pattern with the lack of references. The most represented groups were Coleoptera and Lepidoptera. The group that performed the best with identifications was Coleoptera and it was also the order that provided the most unique BINS, in contrast with Lepidoptera that provided the least unique BINS also outperformed GenBank at the species and genus level, but no differences were found at family.

A great potential of Colombian insect biodiversity was found from BINs counting with 70% of the sequences assigned to a unique BIN. Each BIN represents a cluster of species or an operational taxonomic unit (OTU) that is compared against the database assigning the barcodes to known clusters (BIN) or creating a new cluster (unique BIN) [21]. Unique BINs were found across all the orders of insects and Coleoptera provided the highest count of new BINs followed by Diptera and Hymenoptera, some of the hyperdiverse orders of insects. However, Lepidoptera was the order with highest non-unique BINs. This finding is likely attributable to a great effort of DNA sampling directed to Lepidoptera that displays 4,848 barcodes from the country with 1,474 BINs [35]; in comparison to Coleoptera with 318 sequences in 82 BINs, Diptera 2,646 with 339 BINs and Hymenoptera 2,179 with 240 BINs [35].

Furthermore, our discovery of 500 unique BINs from our dataset of 1,088 sequences showcases the need for further sampling and identification in order to add more species into these databases and increase the accuracy of matches. However, this would require the implementation of a systematic method of collecting samples across Colombia, a process that could take a considerable length of time and amount of labor. Of our seven collecting methods used in this study, the malaise traps accounted for 62.6%, and we would suggest a targeted approach by placing these traps in strategic areas across Colombia. We expect that a larger sample will continue providing a higher number of unique BINs, but also, we see a necessary taxonomic effort to match all of this genetic diversity with the insect species already described from Colombia. Nevertheless, the implementation of DNA barcodes in the country will bring an opportunity to recognize any hidden diversity. As it has been suggested that BINs underestimate by a 10% the species due to lack of power within close species [11, 36, 37]. Although Meier et al. [38] suggested awareness on the use of BINS as identification tool specially when use for species descriptions. Therefore, an integrative approach with morphology and genetic diversity can boost the recognition of species.

A closer look to the subfamily Scarabaeinae displayed a great performance of identification at different levels. The percentage of similarity for a match (BOLD match percentage) to identify species correctly in Scarabaeinae was in a wide range between 93% to 100%. Although correct matches were statistically significant the range was not perfect, as shown at least three specimens were incorrectly identified with values higher than 94%. These outliers do not reduce the potential of genetic identification tools. The high percentages could be due to specimen mix-up. Overall, the identification success rate of Scarabaeinae was 85% (30% towards genera and 55% towards species) when combining the barcode results with a taxonomist effort (Table 3 and Fig 4A). The species identification improved from 78% to 96% (or in the overall from 30% to 55%) with this taxonomist *de novo* identifications (Table 3 and Fig 4A). Other studies of identification of beetles using barcodes found species matches as high as 92.1% of their samples when considered traditionally identified species and BINS and as high as 98.3% of the samples when haplotypes were considered [39]. In comparison, our identification percentage was lower with previous studies. These can be due to the lack of sequence records of the species in the database. Pentinsaari et al. [39] suggested identification failures are due to difficulty with morphological characters and controversial taxonomic status of species. Taxonomic input is crucial for the function of this tool, also if the species is not represented in the database there is no possible match to occur. In fact, a few suggestions provided by BOLD for incorrect species identifications were for close related species or species within their group of species. For example, *Dichotomius andresi* and *Dichotomius protectus* were matched with *Dichotomius satanas*, a morphologically related species in the “*Dichotomius satanas* species group”. Similarly, *Eurysternus hypocrita* was identified as *Eurysternus olivaceus*, a species in the "*Eurysternus velutinus* species group". The specimen identified as *Phanaeus pyrois* was matched as *Phanaeus malyi*, a species that was long considered synonymous with *P*. *pyrois*. Currently, *P*. *malyi* has been revalidated and registered in Colombia without a specific location [40].

The distribution range of species could be an additional factor as observed with the dung beetles data where BOLD identify all species correctly in six genera (*Coprophanaeus*, *Digitonthophagus*, *Malagoniella*, *Onthophagus*, *Phanaeus* and *Sulcophanaeus*), eight genera partially correct with some of the species identified correctly but not all (*Ateuchus*, *Canthidium*, *Canthon*, *Deltochilum*, *Dichotomius*, *Eurysternus*, *Ontherus* and *Uroxys*) and failed completely for the genera *Pseudocanthon* (1 specimen) and *Scybalocanthon* (6 specimens). The correctly identified species were obtained for species with a wide distribution and shared between South America and Central America, such as *Canthon juvencus*, *Canthon subhyalinus*, *Eurysternus caribaeus*, *Eurysternus foedus*, *Eurysternus mexicanus Onthophagus acuminatus*, *Onthophagus lebasi*, and *Uroxys micros*, most likely due to representativeness of COI sequences of these species from Costa Rica [41]. These species are found across countries or ecosystems (e. g. dry and humid forests, natural and disturbed ecosystems). In contrast, the identification was incorrect for some species with restricted distributions in Colombia such as *Canthon arcabuquensis*, *Dichotomius andresi*, *Uroxys cuprescens*, and *Deltochilum susanae*, the latest was first identified as the morphospecies *Deltochilum* sp. 12H [42] and later described as a new species [43] or species with Andean distributions as in *Dichotomius protectus*, *Eurysternus marmoreus* and *Ontherus brevicollis* [44–49]. In the case of Scarabaeinae dung beetles, the barcode results helped to confirm at least five species that were assigned as morphospecies in the dung beetles of Colombia “Reference Collection” hosted at of IAVH Entomological Collection. The diversity of dung beetle’s subfamily Scarabaeinae in Colombia exceeds by a large number of species that of neighboring countries such as Panama, Ecuador or Brazil. In general, a defined and well-identified species in Colombia has other species, morphologically similar and taxonomically closer, that have been assigned as morphospecies. This is why the Reference Collection was created, which so far houses about 85% percent of species of Colombia as morphospecies. We see the barcode as a fundamental integrative taxonomy tool, which will help to outline these morphospecies and define which of those are new species for science, and to improve the final taxonomic list of this important group of beetles. Overall, there are still challenges to overcome and more research is recommended in this topic. The reference databases keep improving and updating their resources and tools for users [23, 34]. BOLD provided reliable identifications for the tested groups of insects overcoming challenges such as a small genetic reference of insects for a highly diverse country.

## Supporting information

S1 Table Project dataset with the insect records. (XLSX)

S2 Table Records of insects by orders with matches from BOLD Batch ID engine and BLAST (GenBank). (XLSX)

S3 Table Chi-squared tests. A) Dataset analyses including BOLD’s first suggestion with the highest percentage match and base pair overlap. This dataset includes matches to sequences on our project. B) Alternative dataset analyses including next higher percentage match and base pair overlap. This excludes matches to sequences from our project. *Is next to orders where Chi-square was significant. **Is next to orders where Chi-square analyzed with Bonferroni correction was significant.
(XLSX)

S4 Table List of Scarabaeinae in other categories as determined by the taxonomist. (XLSX)

## References

[1] Díaz S, Settele J, Brondízio E, Ngo H, Guèze M, Agard J, et al. IPBES (2019): Summary for policymakers of the global assessment report on biodiversity and ecosystem services of the Intergovernmental Science-Policy Platform on Biodiversity and Ecosystem Services. Bonn, Germany: IPBES secretariat; 2019 p. 56. Available: https://www.ipbes.net/

[2] Barnosky AD, Matzke N, Tomiya S, Wogan GOU, Swartz B, Quental TB, et al. Has the Earth’s sixth mass extinction already arrived? Nature. 2011;471: 51–57. doi: 10.1038/nature09678 21368823

[3] CBD. Convention on Biological Diversity. 2019 [cited 2 Sep 2019]. Available: https://www.cbd.int/

[4] Janzen DH, Hallwachs W. Perspective: Where might be many tropical insects? Biol Conserv. 2019;233: 102–108. doi: 10.1016/j.biocon.2019.02.030

[5] Sánchez-Bayo F, Wyckhuys KAG. Worldwide decline of the entomofauna: A review of its drivers. Biol Conserv. 2019;232: 8–27. doi: 10.1016/j.biocon.2019.01.020

[6] IUCN. The IUCN Red List of Threatened Species. 2022 [cited 4 Sep 2022]. Available: https://www.iucnredlist.org

[7] Arbeláez-Cortés E. Knowledge of Colombian biodiversity: published and indexed. Biodivers Conserv. 2013;22: 2875–2906. doi: 10.1007/s10531-013-0560-y

[8] Morinière J, Cancian de Araujo B, Lam AW, Hausmann A, Balke M, Schmidt S, et al. Species Identification in Malaise Trap Samples by DNA Barcoding Based on NGS Technologies and a Scoring Matrix. Fontaneto D, editor. PLOS ONE. 2016;11: e0155497. doi: 10.1371/journal.pone.0155497 27191722 PMC4871420

[9] Arias-Penna DC, Whitfield JB, Janzen DH, Hallwachs W, Dyer LA, Smith MA, et al. A species-level taxonomic review and host associations of Glyptapanteles (Hymenoptera, Braconidae, Microgastrinae) with an emphasis on 136 new reared species from Costa Rica and Ecuador. ZooKeys. 2019;890: 1–685. doi: 10.3897/zookeys.890.35786 31798309 PMC6881475

[10] Sharkey MJ, Janzen DH, Hallwachs W, Chapman EG, Smith MA, Dapkey T, et al. Minimalist revision and description of 403 new species in 11 subfamilies of Costa Rican braconid parasitoid wasps, including host records for 219 species. ZooKeys. 2021;1013: 1–665. doi: 10.3897/zookeys.1013.55600 34512087 PMC8390796

[11] Janzen DH, Hallwachs W, Pereira G, Blanco R, Masis A, Chavarria MM, et al. Using DNA-barcoded Malaise trap samples to measure impact of a geothermal energy project on the biodiversity of a Costa Rican old-growth rain forest. Genome. 2020;63: 407–436. doi: 10.1139/gen-2020-0002 32579871

[12] Geiger M, Moriniere J, Hausmann A, Haszprunar G, Wägele W, Hebert P, et al. Testing the Global Malaise Trap Program–How well does the current barcode reference library identify flying insects in Germany? Biodivers Data J. 2016;4: e10671. doi: 10.3897/BDJ.4.e10671 27932930 PMC5136679

[13] Steinke D, Breton V, Berzitis E, Hebert PDN. The School Malaise Trap Program: Coupling educational outreach with scientific discovery. PLOS Biol. 2017;15: e2001829. doi: 10.1371/journal.pbio.2001829 28437475 PMC5402927

[14] Kutty SN, Wang W, Ang Y, Tay YC, Ho JKI, Meier R. Next-Generation identification tools for Nee Soon freshwater swamp forest, Singapore. Gard Bull Singap. 2018;70: 155–173. doi: 10.26492/gbs70(suppl.1).2018-08

[15] Karlsson D, Hartop E, Forshage M, Jaschhof M, Ronquist F. The Swedish Malaise Trap Project: A 15 Year Retrospective on a Countrywide Insect Inventory. Biodivers Data J. 2020;8: e47255. doi: 10.3897/BDJ.8.e47255 32015667 PMC6987249

[16] Srivathsan A, Lee L, Katoh K, Hartop E, Kutty SN, Wong J, et al. ONTbarcoder and MinION barcodes aid biodiversity discovery and identification by everyone, for everyone. BMC Biol. 2021;19: 217. doi: 10.1186/s12915-021-01141-x 34587965 PMC8479912

[17] Wilson JJ. DNA Barcodes for Insects. In: Kress WJ, Erickson DL, editors. DNA Barcodes. Totowa, NJ: Humana Press; 2012. pp. 17–46. doi: 10.1007/978-1-61779-591-6_3 22684951

[18] Magoga G, Forni G, Brunetti M, Meral A, Spada A, De Biase A, et al. Curation of a reference database of COI sequences for insect identification through DNA metabarcoding: COins. Database. 2022; 1–7. doi: 10.1093/database/baac055 35796594 PMC9261288

[19] Hebert PDN, Cywinska A, Ball SL, deWaard JR. Biological identifications through DNA barcodes. Proc R Soc Lond B Biol Sci. 2003;270: 313–321. doi: 10.1098/rspb.2002.2218 12614582 PMC1691236

[20] Ratnasingham S, Hebert PDN. bold: The Barcode of Life Data System (http://www.barcodinglife.org). Mol Ecol Notes. 2007;7: 355–364. doi: 10.1111/j.1471-8286.2007.01678.x 18784790 PMC1890991

[21] Ratnasingham S, Hebert PDN. A DNA-Based Registry for All Animal Species: The Barcode Index Number (BIN) System. Fontaneto D, editor. PLoS ONE. 2013;8: e66213. doi: 10.1371/journal.pone.0066213 23861743 PMC3704603

[22] Altschul SF, Gish W, Miller W, Myers EW, Lipman DJ. Basic local alignment search tool. J Mol Biol. 1990;215: 403–410. doi: 10.1016/S0022-2836(05)80360-2 2231712

[23] Schoch CL, Ciufo S, Domrachev M, Hotton CL, Kannan S, Khovanskaya R, et al. NCBI Taxonomy: a comprehensive update on curation, resources and tools. Database. 2020;2020: baaa062. doi: 10.1093/database/baaa062 32761142 PMC7408187

[24] Meiklejohn KA, Damaso N, Robertson JM. Assessment of BOLD and GenBank–Their accuracy and reliability for the identification of biological materials. Fugmann SD, editor. PLOS ONE. 2019;14: e0217084. doi: 10.1371/journal.pone.0217084 31216285 PMC6584008

[25] Pentinsaari M, Ratnasingham S, Miller SE, Hebert PDN. BOLD and GenBank revisited–Do identification errors arise in the lab or in the sequence libraries? Kuntner M, editor. PLOS ONE. 2020;15: e0231814. doi: 10.1371/journal.pone.0231814 32298363 PMC7162515

[26] Becker JJ, Sandwell DT, Smith WHF, Braud J, Binder B, Depner J, et al. Global Bathymetry and Elevation Data at 30 Arc Seconds Resolution: SRTM30_PLUS. Mar Geod. 2009;32: 355–371. doi: 10.1080/01490410903297766

[27] González-Alvarado AF, Torres E, Medina CA. Escarabajos coprófagos (Coleoptera: Scarabaeidae: Scarabaeinae) de bosques secos colombianos en la Colección Entomológica del Instituto Alexander von Humboldt. Biota Colomb. 2015;16: 88–95.

[28] Ivanova NV, Dewaard JR, Hebert PDN. An inexpensive, automation-friendly protocol for recovering high-quality DNA: Technical Note. Mol Ecol Notes. 2006;6: 998–1002. doi: 10.1111/j.1471-8286.2006.01428.x

[29] Folmer O, Black M, Hoeh W, Lutz R, Vrijenhoek R. DNA primers for amplification of mitochondrial cytochrome c oxidase subunit I from diverse metazoan invertebrates. Mol Mar Biol Biotechnol. 1994;3: 294–299. 7881515

[30] Hebert PDN, Penton EH, Burns JM, Janzen DH, Hallwachs W. Ten species in one: DNA barcoding reveals cryptic species in the neotropical skipper butterfly *Astraptes fulgerator*. Proc Natl Acad Sci. 2004;101: 14812–14817. doi: 10.1073/pnas.0406166101 15465915 PMC522015

[31] Kearse M, Moir R, Wilson A, Stones-Havas S, Cheung M, Sturrock S, et al. Geneious Basic: An integrated and extendable desktop software platform for the organization and analysis of sequence data. Bioinformatics. 2012;28: 1647–1649. doi: 10.1093/bioinformatics/bts199 22543367 PMC3371832

[32] Hammer O, Harper DAT, Ryan PD. PAST: Paleontological Statistics Software Package for Education and Data Analysis. Palaeontol Electron. 4: 9.

[33] Cupello M, Vaz-de-Mello FZ. A monographic revision of the Neotropical dung beetle genus Sylvicanthon Halffter & Martínez, 1977 (Coleoptera: Scarabaeidae: Scarabaeinae: Deltochilini), including a reappraisal of the taxonomic history of ‘Canthon sensu lato.’ Eur J Taxon. 2018. doi: 10.5852/ejt.2018.467

[34] Madden MJL, Young RG, Brown JW, Miller SE, Frewin AJ, Hanner RH. Using DNA barcoding to improve invasive pest identification at U.S. ports-of-entry. Labra M, editor. PLOS ONE. 2019;14: e0222291. doi: 10.1371/journal.pone.0222291 31527883 PMC6748562

[35] BOLD systems public data portal—record list Database. 2022 [cited 21 Jul 2022]. Available: https://www.boldsystems.org/

[36] Janzen DH, Hallwachs W. DNA barcoding the Lepidoptera inventory of a large complex tropical conserved wildland, Area de Conservacion Guanacaste, northwestern Costa Rica. Genome. 2016;59: 641–660. doi: 10.1139/gen-2016-0005 27584861

[37] Janzen DH, Hallwachs W, Blandin P, Burns JM, Cadiou J-M, Chacon I, et al. Integration of DNA barcoding into an ongoing inventory of complex tropical biodiversity. Mol Ecol Resour. 2009;9: 1–26. doi: 10.1111/j.1755-0998.2009.02628.x 21564960

[38] Meier R, Blaimer BB, Buenaventura E, Hartop E, Rintelen T, Srivathsan A, et al. A re‐analysis of the data in Sharkey et al.’s (2021) minimalist revision reveals that BINs do not deserve names, but BOLD Systems needs a stronger commitment to open science. Cladistics. 2022;38: 264–275. doi: 10.1111/cla.12489 34487362

[39] Pentinsaari M, Hebert PDN, Mutanen M. Barcoding Beetles: A Regional Survey of 1872 Species Reveals High Identification Success and Unusually Deep Interspecific Divergences. Fontaneto D, editor. PLoS ONE. 2014;9: e108651. doi: 10.1371/journal.pone.0108651 25255319 PMC4177932

[40] Moctezuma V, Halffter G. Taxonomic revision of the Phanaeus endymion species group (Coleoptera: Scarabaeidae), with the descriptions of five new species. Eur J Taxon. 2021;747: 1–71. doi: 10.5852/ejt.2021.747.1333

[41] Solís Á, Kohlmann B. Checklist and distribution atlas of the Scarabaeinae (Coleoptera: Scarabaeidae) of Costa Rica. Zootaxa. 3482: 1–32. doi: 10.11646/zootaxa.3482.1.1

[42] Martínez-Revelo DE, Castro-Moreno C, Medina CA. Escarabajos coprófagos de la cuenca alta y media del río Bita, Vichada, Colombia. Biota Colomb. 2018;19: 226–235. doi: 10.21068/c2018.v19n01a15

[43] González-Alvarado A, Vaz-de-Mello FZ. Towards a comprehensive taxonomic revision of the Neotropical dung beetle subgenus Deltochilum (Deltohyboma) Lane, 1946 (Coleoptera: Scarabaeidae: Scarabaeinae): Division into species-groups. Zhang F, editor. PLOS ONE. 2021;16: e0244657. doi: 10.1371/journal.pone.0244657 33406525 PMC7787713

[44] Génier F. A revision of the Neotropical genus Ontherus Erichson (Coleoptera: Scarabaeidae, Scarabaeinae). Mem Entomol Soc Can. 1996;128: 3–170. doi: 10.4039/entm128170fv

[45] Camero Rubio E, Lobo JM. The Distribution of the Species of Eurysternus Dalman, 1824 (Coleoptera: Scarabaeidae) in America: Potential Distributions and the Locations of Areas to be Surveyed. Trop Conserv Sci. 2012;5: 225–244. doi: 10.1177/194008291200500210

[46] Camero Rubio E, Lobo JM. Distribución conocida y potencial de las especies del género Eurysternus Dalman, 1824 (Coleoptera: Scarabaeidae) de Colombia. Bol SEA. 2010; 257–264.

[47] Sarmiento-Garcés R, Amat-García G. Escarabajos del género Dichotomius Hope 1838 (Scarabaeidae: Scarabaeinae) en Colombia. Fauna de Colombia. Monografía 4. Bogotá, Colombia: Universidad Nacional de Colombia; 2014. doi:10.13140/2.1.4692.9926

[48] Medina C, González A. Escarabajos coprófagos de la subfamilia Scarabaeinae. In: Pizano C, García H, editors. El Bosque Seco Tropical en Colombia. Instituto de Investigación de Recursos Biológicos Alexander von Humboldt (IAvH); pp. 195–213.

[49] Chamorro W, Marin-Armijos D, Asenjo A, Vaz-De-Mello FZ. Scarabaeinae dung beetles from Ecuador: a catalog, nomenclatural acts, and distribution records. ZooKeys. 2019;826: 1–343. doi: 10.3897/zookeys.826.26488 30858752 PMC6405737

